# 3D Visualization, Skeletonization and Branching Analysis of Blood Vessels in Angiogenesis

**DOI:** 10.3390/ijms24097714

**Published:** 2023-04-23

**Authors:** Vignesh Ramakrishnan, Rebecca Schönmehl, Annalena Artinger, Lina Winter, Hendrik Böck, Stephan Schreml, Florian Gürtler, Jimmy Daza, Volker H. Schmitt, Andreas Mamilos, Pablo Arbelaez, Andreas Teufel, Tanja Niedermair, Ondrej Topolcan, Marie Karlíková, Samuel Sossalla, Christoph B. Wiedenroth, Markus Rupp, Christoph Brochhausen

**Affiliations:** 1 Institute of Pathology, University of Regensburg, 93053 Regensburg, Germany; 2 Central Biobank Regensburg, University and University Hospital Regensburg, 93053 Regensburg, Germany; 3 Institute of Pathology, University Medical Centre Mannheim, Heidelberg University, 68167 Mannheim, Germany; 4 Department of Dermatology, University Medical Centre Regensburg, 93053 Regensburg, Germany; 5 Department of Internal Medicine II, Division of Hepatology, Medical Faculty Mannheim, Heidelberg University, 68167 Mannheim, Germany; 6 Department of Cardiology, University Medical Centre, Johannes Gutenberg University of Mainz, 55131 Mainz, Germany; 7 Center for Research and Formation in Artificial Intelligence (CinfonIA), Universidad de Los Andes, 111711 Bogota, Colombia; 8 Biomedical Center, Faculty of Medicine in Pilsen, Charles University, 32300 Pilsen, Czech Republic; 9 Department of Internal Medicine II, University Hospital Regensburg, 93053 Regensburg, Germany; 10 Department of Thoracic Surgery, Kerckhoff Clinic, 61231 Bad Nauheim, Germany; 11 Department of Trauma Surgery, University Medical Centre Regensburg, 93053 Regensburg, Germany

**Keywords:** angiogenesis, 3D visualization, neural networks, image registration and segmentation, artificial intelligence, digital pathology, biobanking

## Abstract

Angiogenesis is the process of new blood vessels growing from existing vasculature. Visualizing them as a three-dimensional (3D) model is a challenging, yet relevant, task as it would be of great help to researchers, pathologists, and medical doctors. A branching analysis on the 3D model would further facilitate research and diagnostic purposes. In this paper, a pipeline of vision algorithms is elaborated to visualize and analyze blood vessels in 3D from formalin-fixed paraffin-embedded (FFPE) granulation tissue sections with two different staining methods. First, a U-net neural network is used to segment blood vessels from the tissues. Second, image registration is used to align the consecutive images. Coarse registration using an image-intensity optimization technique, followed by finetuning using a neural network based on Spatial Transformers, results in an excellent alignment of images. Lastly, the corresponding segmented masks depicting the blood vessels are aligned and interpolated using the results of the image registration, resulting in a visualized 3D model. Additionally, a skeletonization algorithm is used to analyze the branching characteristics of the 3D vascular model. In summary, computer vision and deep learning is used to reconstruct, visualize and analyze a 3D vascular model from a set of parallel tissue samples. Our technique opens innovative perspectives in the pathophysiological understanding of vascular morphogenesis under different pathophysiological conditions and its potential diagnostic role.

## 1. Introduction

Angiogenesis represents the formation of new blood vessels from existing vasculature, involving the migration, growth and differentiation of endothelial cells, which line the inner surface of blood vessels. The process plays an integral part in the proliferative stage of wound healing, forming new blood vessels from pre-existing ones by invading the wound clot and organizing into a microvascular network throughout the granulation tissue [1]. Angiogenesis is also a crucial prerequisite for invasive tumor growth and metastasis and constitutes an important point of control with respect to cancer progression [2]. Consequently, it is one of the eight hallmarks of cancer [3]. The detailed processes of forming three-dimensional (3D) vascular networks during morphogenesis are not yet fully understood in detail, even with the help of powerful hybrid mathematical and computational models [4]. To enhance the research in angiogenesis, it is essential to focus on innovative ideas to improve the research into angiogenesis. Specifically, this includes the need for the 3D visualization and analysis of a vascular network.

Recent studies have demonstrated that it is possible to reconstruct objects as a 3D model using a set of parallel consecutive images through image registration and segmentation [5,6]. For medical research, neurons were reconstructed in 3D to study the relationship between cell morphology and function [7]. A human Schlemm’s canal was visualized in 3D to provide diagnostic information in the eye [8]. Furthermore, with the availability of 3D data points, several other applications are possible in the medical domain. For example, a quantitative characterization of the vascular network was studied with the help of skeletonization on 3D photoacoustic images [9]. Skeletonization on a 3D model also aids in path finding in the virtual endoscopy and analysis of 3D pathological sample images [10].

The challenge of this paper is the concept of image registration to align images and image segmentation to isolate blood vessels to build and reconstruct a 3D model. To understand the specifics of the overall pipeline, the region of interest (ROI) to be visualized is selected by a pathologist. The first step involves using an image segmentation algorithm to segment the blood vessels as relevant objects in the specified ROI. The next step is to ensure the strict alignment of consecutive images using image registration. The corresponding segmented masks are aligned using the results of the registration algorithm and are interpolated to reconstruct and visualize a 3D model. For analysis, a skeleton model is derived from the 3D reconstructed model, with which the branching of blood vessels could be analyzed. The next section elaborates on the relevant state-of-the-art algorithms used and the basis for choosing them.

The aim of the present report is to demonstrate a pipeline of computer vision and deep-learning-based algorithms to accurately visualize blood vessels in 3D from a set of consecutive tissue samples.

## 2. Results

**Figure 1:**
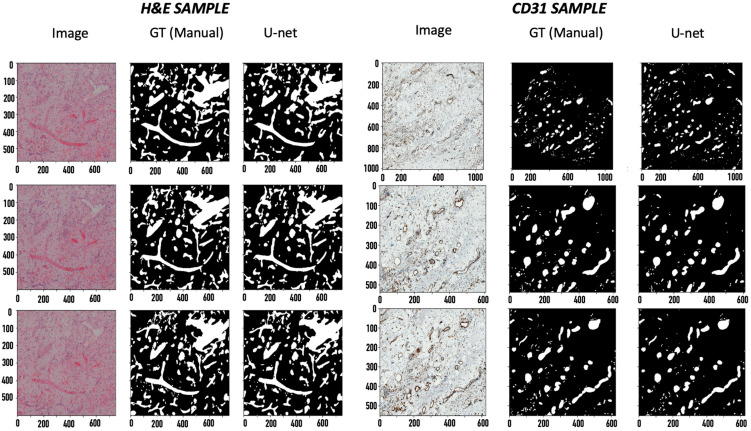
Segmentation results. 1. Image, 2. GT, 3. U-net predicted mask. The x axis applies to the respective column. The values are specified in pixels (magnification: 20× for H&E and 40× for CD31).

After training the tissue samples with 70% of the patched Ground-Truth (GT) data via the U-net segmentation network [11] to a validation dice score of greater than 90%, dice score accuracies of 92.1% and 91.7% were observed on the H&E-stained tissues and CD31-stained tissues, respectively (Figure 1). The dice score accuracy using the U-net segmentation algorithm is compared to the traditional block-based Otsu method (Table 1).

**Figure 2:**
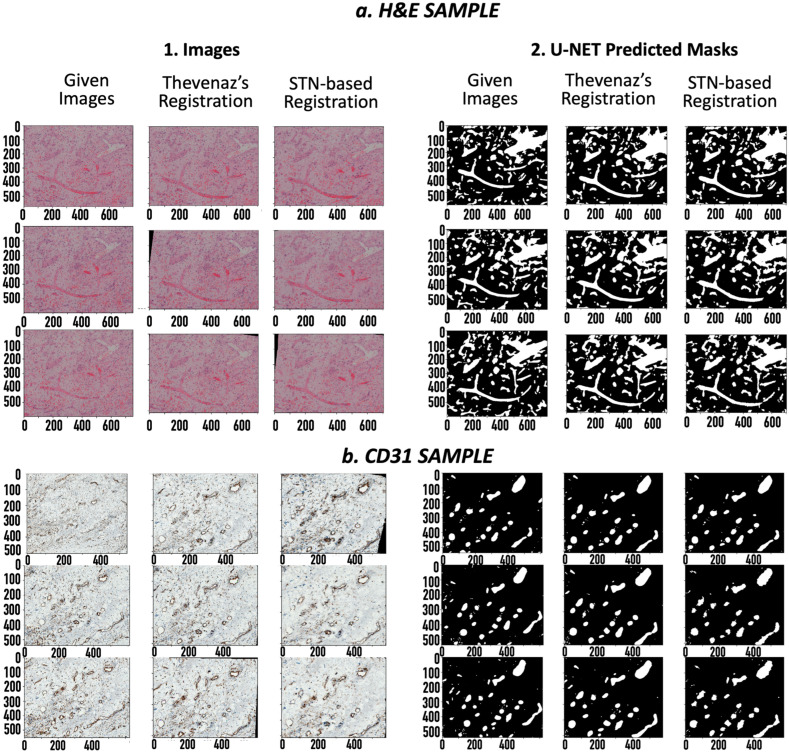
Coarse-to-fine registered images: (**a1**). H&E images (20× magnification), (**a2**). H&E masks, (**b1**). CD31 images (40× magnification), (**b2**). CD31 masks. For each set, left to right: i. Original image/mask, ii. Coarsely registered image/mask using Thevenaz’s algorithm, iii. Finely registered images/masks using spatial transformers. The x axis applies to the respective column. The values are specified in pixels.

After coarse image registration applying Thevenaz’s algorithm [13], the SSIM score for the H&E sample was improved from 29.2% to 46.3%, and for the CD31 images, from 9.1% to 14.9%. A significant leap was seen in the mutual dice scores for the corresponding binary GT masks of the H&E samples and CD31 samples, from 3.8% to 31.8% and 0% to 14.3% (Table 2), respectively. Finetuning the registration algorithm using the STN-based registration network [14] further increased the SSIM score to 57.0% for H&E and 25.6% for CD31. Also, the mutual dice score on the corresponding GT masks improved to 32.4% for H&E and 18.2% for CD31. The mutual dice metrics on the predicted masks reciprocated the results for both the H&E (Table 3) and CD31 samples (Table 4). A visual overview of the images and their corresponding masks before and after image registration gives us an idea of the extent of the achieved image registration (Figure 2).

**Figure 3:**
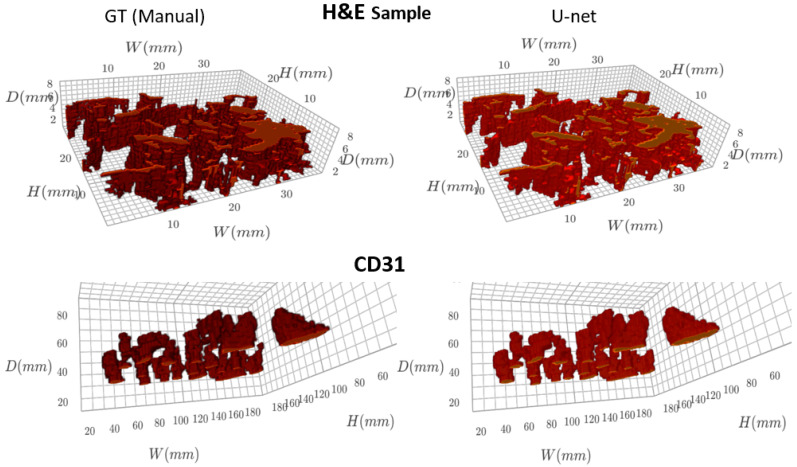
Visualizing the 3D model of blood vessels (GT and U-net predicted masks).

**Figure 4:**
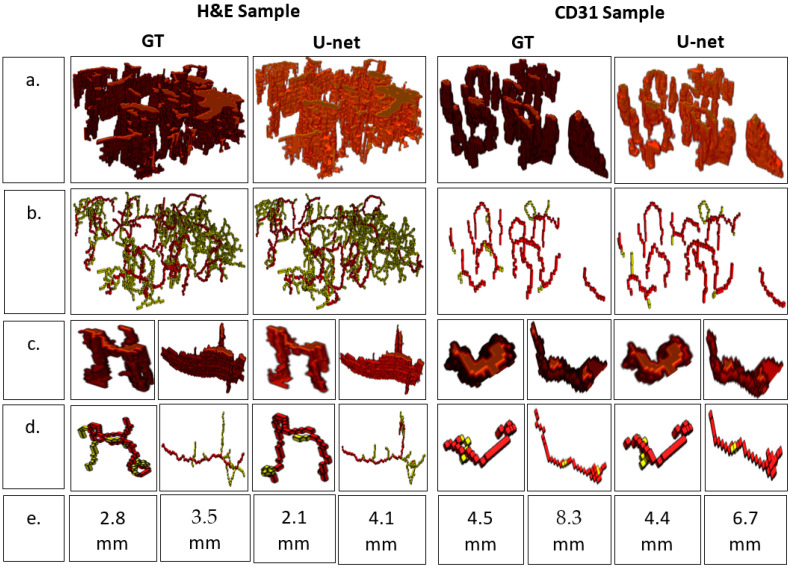
Analyzing branch statistics of the 3D models. (**a**) 3D model, (**b**) Skeletonized model highlighting the main branch for each individual blood vessel, (**c**) 3D models of individual blood vessels, (**d**) 3D skeletonized model of individual blood vessels, (**e**) Main branch length (mm).

The finely registered binary masks, both the GT and the predicted vessels, were interpolated to the tissue dimensions using bilinear interpolation [15], and a 3D model was reconstructed (Figure 3). A video of the reconstructed 3D models is available in the Supplementary Material. The resulting 3D models were skeletonized using Lee’s skeletonization algorithm [16]. Subsequently, it was possible to identify and evaluate the branching statistics. It was also possible to find the length of the main branch from the skeletonized 3D model (Figure 4).

## 3. Discussion

Image registration and segmentation are the two most significant steps of this pipeline, followed by image interpolation and skeletonization algorithms. Several approaches in semi-supervised image segmentation, including the well-known U-net segmentation algorithm [11], are reported in the literature, which produce excellent results for the task of image segmentation [17]. In contrast, unsupervised image registration is an extremely challenging task to accomplish, with much more scope for research. The two techniques used in our work are known to be among the most reliable algorithms for coarse-to-fine image registration [18,19], resulting in excellent 3D models.

The reliability of image segmentation can be easily estimated using dice score accuracies, which provide a measure of the amount of overlap between the automatically predicted annotations and the GT annotations. The observed dice scores of 92.1% and 91.7% on the two samples show the excellent reliability of the deep-learning-based U-net segmentation network. Moreover, comparing it to the results of a simple block-based Otsu approach shows a difference of 25–35% in the dice score accuracy (Table 1), which reinforces the dominance of deep learning algorithms for image segmentation. The reliability of an image registration algorithm is difficult to estimate as image registration is an unsupervised algorithm, and it is not feasible to manually register images. However, the proposed analysis, using similarity scores (SSIM) and the mutual dice score, provides a reasonable justification to show the reliability of the image registration algorithms on the given set of images. This can evidently be seen by visually viewing the images before and after the registration for the two samples, where inconsistencies in the rotation and translation were corrected (Table 2 and Figure 1). The increase in the SSIM using coarse and fine registration showed that the given set of misaligned images were aligned (Table 2). Interestingly, the corresponding dice score accuracies for the consecutive images provided even stronger evidence of image alignment. The dice score accuracies for the consecutive image pairs increased from ~30% for the unregistered images to ~80% after registration (Table 3 and Table 4). Considering that the consecutive images need to be as close to each other as possible, the high dice scores provide strong grounds to suggest excellent registration results. Moreover, the mutual dice score, which indicates the amount of overlap across the complete set of images, increased from ~0% for the misaligned images to a significant value (~35% for the H&E samples and ~18% for the CD31 samples). Thus, we provided strong evidence that the given set of images are reliably aligned, making the corresponding masks suitable for 3D visualization. Therefore, the aligned images provided a meaningful 3D model of the blood vessels.

The main purpose of this work is to introduce a reliable pipeline to reconstruct a 3D model of blood vessels from a set of parallel slides of granulation tissue samples by employing algorithms based on computer vision and deep learning. Reconstructing vessels manually in 3D is very tedious and requires a lot of time and manual labor. An automatic approach would help pathologists to improve the research and diagnostic potency of structural defects in the vascular network or during angiogenesis, respectively.

The successful reconstruction of a 3D model from two independent tissue samples with completely different staining techniques (H&E and CD31) implies that the type of staining has no effect on the reconstruction. Similarly, the type of tissue plays no role if it is possible to obtain parallel slides of the tissue. As an example, one could use this pipeline to reconstruct blood vessels from endomyocardial tissue from several heart diseases [20,21]. In this context, the ability to reuse heart tissues from biobanks [22] for 3D reconstruction could be a future research approach. Furthermore, our pipeline could also be used for the 3D reconstruction of tumor vessels in cancer patients to better understand the morphogenesis of the vasculature in invasive tumor masses.

The 3D model of blood vessels could also be used for various applications in analyzing wound healing or wound healing deficiency, respectively. In addition, this model could be used to verify mathematical models describing the geometry of blood vessels in angiogenesis [4].

A branching analysis of the 3D model was possible by post-processing the 3D model using the skeletonization algorithm devised by Lee [16]. The skeletal 3D model was analyzed using the Skan library [23], which focuses on finding individual branches of a skeletal model. As a result, a visualization model of individual blood vessels was achieved, and the length of the branches could be estimated for the individual blood vessels in the 3D model (Figure 2).

There are limitations to this approach. Firstly, obtaining these stained images involves tissue destructive histological methods. Secondly, the tissues need to be carefully stained for a reliable 3D reconstruction. This could otherwise lead to false results as the algorithm could converge to a local minimum. This challenge can be overcome through manual intervention, by choosing selected regions to run the algorithms. Lastly, the mutual dice score relies on the assumption that the blood vessels follow a continuous flow structure. For a large number of consecutive samples, the blood vessels could gradually flow out of the considered frame. For such cases, it could be better to consider the mutual dice score for overlapped batches of consecutive images for reliable registration.

For future research, the use of Generative Adversarial Networks (GANs) [24] could be investigated for even better registration results [25]. In contrast, a stable convergence is difficult and time consuming, even with the availability of high computational hardware, along with the risk of the introduction of undesirable artifacts using GANs. The currently used bilinear interpolation gives reasonable accuracy to visualize the model. Nonetheless, research in deep learning approaches to performing interpolation would further improve the visualization, resulting in an even better 3D model. Another area to focus on is the use of vision-based approaches in radiomics. There has already been some progress in using artificial intelligence in radiomics, which is a novel approach for solving precision methods using non-invasive, low-cost and fast multimodal medical images [26]. The application of the random forests algorithm was studied to predict the prognostic factors of breast cancer by integrating tumor heterogeneity and angiogenesis properties on MRI [27]. Low-field nuclear magnetic resonance (NMR) relaxometry, with the aid of machine learning, could rapidly and accurately detect objects [28]. The speed of such algorithms has been improved, as well using low-field NMR relaxometry [29]. Although the results obtained by algorithms on non-invasive, low-cost images would not be as reliable as images obtained through invasive methods, it would be interesting to understand the available data from radiomics and to analyze the applications of vision-based algorithms in the future.

## 4. Materials and Methods

### 4.1. Tissue Preparation and Data Acquisition

Our experiments were performed on two samples of human granulation tissue. The first sample was a set of eight consecutively sliced tissue sections of H&E-stained granulation tissue. The second one was a set of ten consecutive tissue sections of a CD31-stained granulation tissue. The thickness of the sections was 4 µm for the H&E and 3 µm for the CD31 samples. For both staining methods, the fresh tissue was first fixed in 5% formalin and then embedded in paraffin. The formalin-fixed paraffin-embedded (FFPE) tissue blocks were cut into consecutive sections and mounted onto glass slides. For the H&E staining, the tissue was deparaffinized, rehydrated and then stained in Mayer’s hematoxylin and eosin. The immunohistochemical staining of CD31 was performed with the help of a BenchMark Ultra autostainer (Ventana Medical Systems, Oro Valley, AZ, USA) employing a mouse anti-CD31 antibody (1:40; clone JC70A; Dako, Glostrup, Denmark). After staining both the H&E and CD31, the slides were covered with glass cover slips and were scanned as whole slide images (WSI) via the PreciPoint M8 slide scanner (PreciPoint GmbH, Freising, Germany) with a 20× objective (Olympus UPlan FLN 20×, Olympus, Tokyo, Japan). The resolution observed at maximum zoom was 0.28 µm per pixel.

### 4.2. Region of Interest (ROI) Selection

The acquired whole slide images were stored in Omero [30], an image server that can be used to view images and store annotations. A particular relevant ROI, where the vessels need to be visualized as a 3D model, was chosen in each consecutive image for the given samples. The chosen ROIs were around 0.5 × 0.4 mm for the H&E samples and 1.3 × 1.2 mm for the CD31 samples. The blood vessels inside the ROI were manually annotated by a medical expert. The manual annotations are referred to as Ground-Truth data (GT). The GT annotations were used to generate a GT mask for semi-supervised segmentation algorithms. They were also used to compare the statistics of the reconstructed 3D model of the GT data and the algorithm-predicted data. Additionally, several metrics to validate the algorithm performance were evaluated using the GT annotations. Consequently, two datasets were generated, which acted as the inputs to the pipeline of the algorithms for the 3D visualization and analysis.

### 4.3. Image Segmentation

Image segmentation is an approach used to partition an image into multiple image segments. The popular traditional approaches, such as Otsu’s method [12], k-Means clustering [31] and the watershed algorithm [32], involve the design of complex hand-crafted features. Although these approaches are unsupervised and computationally efficient, the design of specific hand-crafted features requires extensive pre-processing, and the resulting accuracy of the segmented models is relatively low [33].

On the contrary, recent advances in deep learning have provided a framework to segment images with extremely high accuracy [34]. With advances in improving deep learning architecture using residual layers [35] and inception layers [36], there has been significant progress in improving deep-learning-based segmentation models [17]. A popular architecture extensively used for medical research is the U-net architecture [11], which uses deconvolution layers and skip-connections to improve the segmentation accuracy [37].

Therefore, a U-net-based segmentation network was chosen to perform image segmentation on blood vessels for our research. The GT data were used for training the semi-supervised algorithm. The dataset was converted into a patched dataset with a patch size of 224 × 224 pixels. For training, the patched dataset was split into 70% training dataset and 30% validation dataset. The network was then trained using an AdamW optimizer [38] with a OneCycleLR scheduler [39] by minimizing the cross-entropy loss. The trained model could then be used to segment blood vessels for the given dataset.

### 4.4. Image Registration

Image registration is a process in which different images are transformed into the same coordinate system by matching the image contents [40]. A broad category of transformation models includes linear transformations involving rotation, scaling, translation, and shear and non-linear transformations comprising elastic, nonrigid or deformable transformations [41,42]. Image registration is extensively used in medical research for several imaging techniques, such as X-ray, CT scans and MRI [43]. Factors such as non-linear geometric distortions, noisy image data and computational complexity contribute to the fact that image registration is one of the most challenging tasks in computer vision [44].

A variety of classical algorithms, which are based on hand-crafted features for image registration, can be used to perform image registration. They are broadly classified into feature-based methods and area-based methods [18]. Feature-based methods extract salient features, such as edges or corners, to align images with the presumption that they stay at fixed positions during the whole experiment [44]. Area-based methods use metrics such as cross-correlation to match image intensities, without performing any structural analysis [32]. An area-based method by Thevenaz et al., which maximizes mutual information using the Marquardt-Levenberg method, has gained tremendous popularity in classical image registration for biomedical research, especially due to its computation-efficient hierarchical search strategy applying a pyramidal approach [13].

Due to its popularity, this paper uses Thevenaz’s algorithm to coarsely register the given dataset. To align the images and remove inconsistencies caused by translation, scale, rotation or shear, Thevenaz’s algorithm [13] was used. The mutual information was minimized between consecutive image pairs to result in a set of affine matrices. The images need to be transformed using the resulting affine matrices through homogeneous transformations. One might need to pre-process the choice of the rectangular sections of the image pair to ensure a reliable coarse alignment and avoid converging to a local minimum. After using the coarse registration approach, images are aligned to a single coordinate system.

### 4.5. Image Registration Finetuning

In the last few years, deep learning has significantly contributed to more accurate image registration using unsupervised transformation estimation methods, especially to predict a deformable transformation model [19]. In particular, Kuang et al. [45] demonstrated excellent results on public datasets using a convolutional neural network (CNN)- and a Spatial Transformer Network (STN)-inspired framework [14] for deformable image registration. This was achieved with the help of a normalized Cross-Correlation (NCC) loss function. Yan et al. [46] showed that a Generative Adversarial Networks (GANs)-based [24] network could estimate rigid transformations. Although GANs are effective in image registration, they are very unstable and prone to synthetic data.

Consequently, to solve non-linear and geometric distortions, an unsupervised deep-learning-based image registration network with a U-net architecture [11] using STNs [14] was used to finetune the coarsely registered images. Pairs of consecutive images were passed as inputs through a neural network for deformable registration via a voxelmorph framework [47]. Flow vectors, which describe the flow of each pixel in the moving image, were predicted using the trained spatial transformers for better registration. To train the network, an AdamW optimizer with a OneCycleLR scheduler was used for smart convergence. A bidirectional loss function based on NCC loss [47], which encourages accurate forward and backward flow vectors, was minimized. A gradient loss was also defined to control the values of the flow vector. After training, the relevant forward flow vectors could be predicted and applied to the coarsely registered stack of images and its corresponding masks, resulting in a finely registered dataset.

### 4.6. Interpolation and 3D Visualization

Image segmentation predicts blood vessels as binary masks. Image registration results in affine matrices and flow vectors to transform and align the given set of unaligned images. The corresponding binary masks can also be aligned using this information, resulting in a sequence of aligned binary masks, which gives us a set of 3D points of blood vessels.

Interpolation is a method to estimate or find new data points based on a range of discrete sets of known data points [48]. A Nearest-Neighbor interpolation [49] introduces significant distortion in the model. A bilinear interpolation [15] gives a better approximation of the 3D model, but the image contours become fuzzy and the high frequency components are faded. A bicubic interpolation [50] would require large amounts of calculation [51].

As a compromise, a bilinear interpolation is chosen for interpolation in our pipeline. The set of 3D points is interpolated, corresponding to the original dimensions of the tissue using bilinear interpolation [15], resulting in the final 3D model. A 3D model is obtained for both the manually annotated (GT) masks and the predicted masks. As a postprocessing step, small irrelevant objects in the 3D models are filtered out. The final models can be visualized and rendered using visualization libraries such as K3D [52] or 3D Slicer [53].

### 4.7. Skeletonization and Analysis

Skeletonization reduces binary objects to a one-pixel-wide representation. This provides a compact representation of the image by reducing the dimensionality to a skeleton and is useful for analyzing the topology and structure of the object. Several skeletonization approaches exist in the literature [54]. Blum and Nagel [55] established the foundation of skeletonization using a Blum’s Grassfire transformation to find a medial axis. After several adaptations, Lee et al. [16] proposed a popular digital approach to simulate Blum’s algorithm by applying a constrained iterative erosion process.

To visualize the structure and flow of blood vessels, a 3D skeletal model of the 3D blood vessel model was obtained using Lee’s skeletonization algorithm [16] from the scikit-image library [56]. With the help of Skan [23], a python library for skeletal analysis, the branch statistics of the skeletal model, was computed. This included the length of the main branch and the number of sub-branches for each blood vessel, along with their lengths. It was also possible to reconstruct a branched skeletal 3D model for visualization.

### 4.8. Metrics

The results of the image registration were analyzed using a Structural Similarity Index Measure (SSIM) [57], which helps to predict the perceived quality of two images. SSIM considers image degradation as a perceived change in the structural information, along with other properties of an image, such as luminance and contrast. An SSIM value of 0 indicates no structural similarity and a value of 1 is only reachable for two identical images. The SSIM value was determined across pairs of images, as well as for the complete sample before and after registration.

The image segmentation was evaluated by calculating the Sorensen-dice coefficient [58,59] or dice score on the segmented vessels. The dice score can be interpreted as a measure to estimate the overlap between binary masks. The GT vessels can be compared to the predicted vessels using this dice score. A dice score of 100 (in percentage) means that the predicted mask perfectly overlaps with the target mask. Mathematically, it can be formulated as:

(1) $$Dice\left(X,Y\right)\left(\%\right)=\frac{2\times \left(X\cap Y\right)}{X\cup Y}\times 100$$

where X and Y are the two masks to be compared.

The dice score can also be used to evaluate the metrics after image registration. It can be computed across two consecutive binary masks after registration. Similarly, the dice score can also be evaluated across the complete sequence, using the following expression:

(2) $$Dice\left(X1,X2,\ldots ,Xn\right)\left(\%\right)=\frac{n\times \left(X1\cap X2\cap \ldots \cap Xn\right)}{X1\cup X2\cup \ldots \cup Xn}\times 100$$

where X1, X2, …, Xn are the binary masks for each image in an image stack. A measure of overlap between the vessels was estimated between the parallel images in an image stack.

## 5. Conclusions

In conclusion, a pipeline of vision-based algorithms, mainly comprising image registration and segmentation, is proposed to visualize and analyze blood vessels as a 3D model. The branching of vasculature could be analyzed using a skeletonization algorithm on the resulting 3D model. Computer vision and deep learning have huge potential to accelerate research in medicine. At present, there is a huge gap between the current developments and the potential of vision-based algorithms. In future studies, we will bridge this gap to accelerate the research in angiogenesis through the use of a 3D model of blood vessels.

## Figures and Tables

**Table 1:** Image segmentation results.

Sample	Block-Based Otsu [12] (Dice Score %)	U-Net Based Network [11] (Dice Score %)
H&E	67.5	92.1
CD31	56.1	91.7

**Table 2:** Coarse-to-fine image registration.

Image Registration	H&E Sample			CD31 Sample		
	Original	Thevenaz’s Algorithm [13]	STN [14]	Original	Thevenaz’s Algorithm [13]	STN [14]
SSIM %	29.2	46.3	57.0	9.1	14.9	25.6
Dice % (GT)	3.8	31.8	32.4	0.0	14.3	16.5
Dice % (U-net)	3.4	34.0	34.9	0.0	16.0	18.2

**Table 3:** Pair-wise image registration for H&E sample.

H&E Sample	Before Registration			After Registration		
	SSIM %	Dice % (GT)	Dice % (U-Net)	SSIM %	Dice % (GT)	Dice % (U-Net)
pair 0, 1	26.0	38.2	38.0	43.4	77.6	80.7
pair 1, 2	28.3	43.3	43.0	45.8	76.4	80.5
pair 2, 3	28.3	43.8	44.0	44.9	79.3	81.4
pair 3, 4	29.5	40.0	40.6	47.9	78.6	81.7
pair 4, 5	33.0	32.5	31.3	50.5	76.3	80.3
pair 5, 6	32.0	33.5	31.3	48.6	76.8	79.8
pair 6, 7	27.6	39.9	39.5	43.3	74.0	75.6
**Full Sequence** **(Avg SSIM % or Mutual Dice %)**	**29.2**	**3.8**	**3.4**	**57.0**	**32.4**	**34.9**

**Table 4:** Pair-wise image registration for CD31 sample.

CD31 Sample	Before Registration			After Registration		
	SSIM %	Dice %(GT)	Dice % (U-Net)	SSIM %	Dice % (GT)	Dice % (U-Net)
pair 0, 1	8.5	25.5	24.8	34.2	80.8	83.5
pair 1, 2	11.4	55.8	55.7	24.2	79.9	81.1
pair 2, 3	11.4	9.7	9.0	20.2	69.7	73.6
pair 3, 4	8.9	9.1	8.0	20.4	71.8	74.9
pair 4, 5	8.6	28.4	28.0	24.7	69.9	70.0
pair 5, 6	8.0	8.8	7.9	24.9	75.8	77.3
pair 6, 7	8.3	38.6	39.0	26.5	74.8	78.3
pair 7, 8	8.1	9.5	12.3	31.4	76.8	78.2
pair 8, 9	8.5	3.4	4.5	24.2	67.5	69.1
**Full Sequence** **(Avg SSIM % or Mutual Dice %)**	**9.1**	**0.0**	**0.0**	**25.6**	**16.5**	**18.2**

## Data Availability

The data presented in this study are openly available in the Supplementary Materials.

## Supplementary Materials

The following supporting information can be downloaded at: https://figshare.com/collections/Data_Angeogenesis_3D_Reconstruction/6339176 (accessed on 20 April 2023).

## Abbreviations

3D: three dimensional, FFPE: formalin-fixed paraffin embedded, H&E: hematoxylin & eosin, CNN: convolutional neural network, STN: Spatial Transformer Network, GAN: Generative Adversarial Networks, GT: Ground-Truth, SSIM: Structural Similarity Index Measure, NCC: normalized Cross-Correlation, NMR: nuclear magnetic resonance, ROI: Region of Interest.
